# Electrified hydrocarbon-to-oxygenates coupled to hydrogen evolution for efficient greenhouse gas mitigation

**DOI:** 10.1038/s41467-023-37382-3

**Published:** 2023-04-07

**Authors:** Wan Ru Leow, Simon Völker, Raoul Meys, Jianan Erick Huang, Shaffiq A. Jaffer, André Bardow, Edward H. Sargent

**Affiliations:** 1 Department of Electrical and Computer Engineering, University of Toronto, 10 King’s College Road, Toronto, Ontario M5S 3G4 Canada; 2 Institute of Sustainability for Chemicals, Energy and Environment (ISCE2), Agency for Science, Technology and Research (A*STAR), 1 Pesek Road, Jurong Island, Singapore 627833 Singapore; 3 Institute of Technical Thermodynamics, RWTH Aachen University, Schinkelstr. 8, 52062 Aachen, Germany; 4 Carbon Minds GmbH, Eupener Straße 165, 50933 Cologne, Germany; 5 TOTAL American Services Inc., Hopkinton, MA 01748 USA; 6 Energy & Process Systems Engineering, Department of Mechanical and Process Engineering, ETH Zürich, 8092 Zürich, Switzerland; 7 Institute of Energy and Climate Research - Energy Systems Engineering (IEK-10), Forschungszentrum Jülich GmbH, 52425 Jülich, Germany

**Keywords:** Electrocatalysis, Environmental health

## Abstract

Chemicals manufacture is among the top greenhouse gas contributors. More than half of the associated emissions are attributable to the sum of ammonia plus oxygenates such as methanol, ethylene glycol and terephthalic acid. Here we explore the impact of electrolyzer systems that couple electrically-powered anodic hydrocarbon-to-oxygenate conversion with cathodic H_2_ evolution reaction from water. We find that, once anodic hydrocarbon-to-oxygenate conversion is developed with high selectivities, greenhouse gas emissions associated with fossil-based NH_3_ and oxygenates manufacture can be reduced by up to 88%. We report that low-carbon electricity is not mandatory to enable a net reduction in greenhouse gas emissions: global chemical industry emissions can be reduced by up to 39% even with electricity having the carbon footprint per MWh available in the United States or China today. We conclude with considerations and recommendations for researchers who wish to embark on this research direction.

## Abstract (web summary)

The production of hydrogen, and the refining of hydrocarbons, are significant contributors to the CO_2_ emissions of the chemicals industry. Coupled electrification of these processes has the potential to reduce total emissions by up to 39% even when using the electricity mix available today.

## Introduction

Chemicals manufacture is among the top greenhouse gas (GHG) contributors, at 18% of global industrial emissions^1^. Of these emissions of chemicals, 85% arise from the large consumption of fossil-based energy and feedstocks, while 15% are direct emissions from the imperfect selectivity of present-day thermochemical production methods: a significant proportion of the hydrocarbon feedstock is oxidized all the way to carbon dioxide (CO_2_) instead of to the desired, partially-oxidized product^2^. Thus, to turn fully carbon-neutral, it is not sufficient simply to switch the energy source from fossil fuels to renewable energy: today’s processes must be replaced by alternatives that do not oxidize hydrocarbons all the way to CO_2_.

Considering all chemicals, more than 50% of GHG emissions are attributable to the sum of ammonia (NH_3_) plus oxygenates such as methanol, ethylene oxide, ethylene glycol, propylene oxide, phenol, and terephthalic acid, which contain oxygen as part of their chemical structure^2^. Dedicated research on new processes to manufacture these chemicals can therefore achieve major impact in reducing net GHG emissions.

**Fig. 1:**
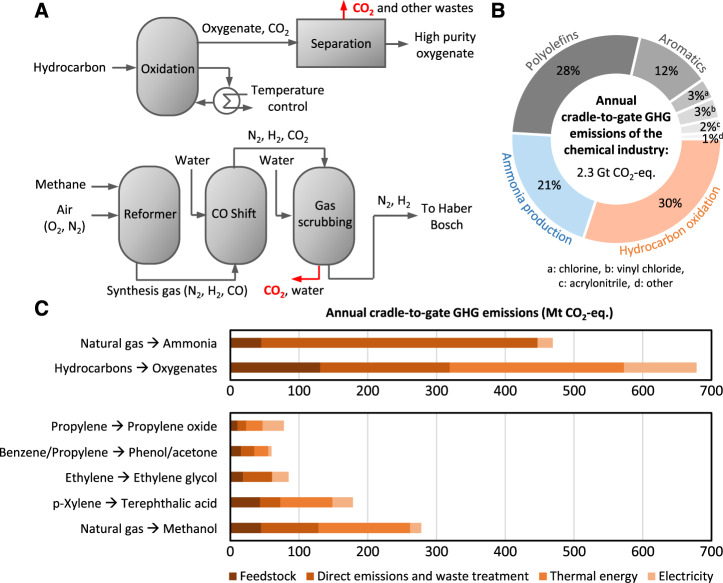
Global climate impact of chemicals manufacture. **A** Conceptual schematic of the thermocatalytic hydrocarbons-to-oxygenates process (detailed present-day processes can be found in Fig. S1–6), as well as H_2_ production via methane reforming and water-gas-shift reactions. The red arrows indicate the sources of direct emissions in Fig. 1C. **B** Annual cradle-to-gate greenhouse gas emissions of the chemical industry in 2030^8,49,55–57^. **C** Breakdown of annual cradle-to-gate emissions of NH_3_ manufacture and hydrocarbon oxidations into feedstock, direct emissions and waste treatment, thermal energy, and electricity.

The production of oxygenates via partial oxidation of hydrocarbons such as ethylene, propylene, and p-xylene is important for the plastics and textiles industries. These processes typically occur at high temperatures and pressures to activate the inert hydrocarbons for functionalization. Due to the exothermic nature of these reactions, extensive cooling is also required to suppress thermal runaway and to minimize the complete oxidation of the hydrocarbons to CO_2_ which limit selectivities of target oxygenates; for example, only about 80% of ethylene will be incorporated into the final product ethylene oxide, with the remaining converted to direct CO_2_ emissions (Fig. 1A)^3^.

Moving now to reduction reactions, NH_3_ manufacture is the largest single contributor to the global GHG emissions of the chemical industry^2^. This is due to the high production volume of NH_3_: over half of global food production relies on ammonia-based fertilizers. The bulk of the GHG emissions stem directly from methane reforming and water-gas-shift reactions to produce the hydrogen (H_2_) feedstock (Fig. 1A).

Despite the existence of a cleaner alternative—water electrolysis to H_2_ and oxygen (O_2_)—less than 2% of the global H_2_ demand is fulfilled this way^4,5^. This is in part because the oxygen evolution reaction (OER) at the anode of water electrolyzers adds considerably to the energy cost of H_2_ + O_2_^6^. Today’s state-of-art water electrolyzers consume about 180 MJ of energy per kg H_2_^7^, which is significantly higher than its lower heating value (LHV) of 120 MJ per kg H_2_. With present-day price of electricity, water electrolysis is therefore more expensive than steam methane reforming^7^. Water electrolysis needs to be powered using low-carbon electricity to achieve a net reduction in GHG emissions. Such electricity is still limited in most industrialized countries today, as electricity from renewable sources such as solar and wind are intermittent and not yet widely available. We focused therefore on strategies that take into account the high energy demand of water electrolysis, and that seek to reduce GHG emissions without relying on ~100%-renewable electricity.

Here we propose that the electrification of hydrocarbon-to-oxygenate conversions can make a significant impact in reducing the carbon footprint of the chemicals industry, due to the high production volumes and GHG emissions. The main intent of the present paper is to evaluate electrolyzer systems that couple anodic hydrocarbon-to-oxygenate conversion with the cathodic H_2_ evolution reaction (HER) from water under ambient conditions. We find that by redirecting the energy that would otherwise be consumed in low-value O_2_ evolution reaction (OER), and instead synthesizing higher-value oxygenates, the approach maximizes the utilization of electricity in producing valuable chemicals. We evaluate, through a prospective life cycle assessment (LCA), the GHG reduction potential compared to thermocatalytic processes. This strategy differs from some prior LCA works which have focused on carbon capture and utilization technologies to reduce emissions associated with the chemicals industry by closing the carbon cycle^8–11^. The present LCA model provides a lower bound for carbon emissions showcasing the best-case scenario when the coupled electrolyzer systems are implemented. It can be seen that, once anodic hydrocarbon-to-oxygenate conversion is developed to target higher selectivities, GHG emissions associated with fossil-based NH_3_ and oxygenates manufacture can be reduced by up to 88%. Low-carbon electricity is not mandatory to enable a net reduction in GHG emissions: indeed, we find that global chemical industry emissions could be reduced by up to 39% even with electricity of carbon intensities (defined as the carbon footprint per MWh electricity) available in the United States and China today^12^. Taking into account regional production facilities, ~18% of global chemical sites currently conduct both NH_3_ manufacture and partial oxidations of hydrocarbons to oxygenates at the same site^13^. These sites can be targeted for implementation of coupled electrolyzer technologies. We close by discussing the gap between industrial needs and present-day electrochemical studies, as well as how the research community can develop electrocatalysts and reaction conditions to realize these new electrolyzer technologies.

## Results

### Global climate impact of NH_3_ and oxygenates manufacture

We evaluate the global climate impact of chemicals manufacture using an LCA-based optimization model that comprises over 400 Life Cycle Inventory (LCI)-compliant datasets of best available technologies for large-volume chemicals and plastics (see Supplementary Materials for details of the LCA model and datasets). The optimization model seeks to find the lowest-carbon avenue based on the available technologies and generates technically feasible energy and material flows throughout the chemical supply chains. These energy and material flows are based on different processes available to produce the desired chemicals. In this publication, the model is used to represent the expected production of 20 large-volume chemicals by 2030. These large-volume chemicals include ammonia, aliphatic hydrocarbons (ethylene, propylene), aromatic hydrocarbons (benzene, styrene, cumene, toluene, p-xylene, and mixed xylenes), oxygenates (methanol, ethylene glycol, ethylene oxide, propylene oxide, terephthalic acid, phenol), polyolefins (polyethylene, polypropylene) as well as caprolactam, acrylonitrile and vinyl chloride (Table S2). In fact, these 20 large-volume chemicals have previously been reported to be responsible for >75% of global GHG emissions of the chemical industry^9^. We therefore use the result as a proxy for the chemical industry. The calculated cradle-to-gate GHG emissions include emissions from the provision of feedstock (i.e., ethylene for ethylene oxide production), thermal energy (i.e., energy needed to power separations and other equipment), electricity (i.e., energy needed for pumps and other equipment) as well as direct emissions and waste treatment (i.e., CO_2_ during steam methane reforming for ammonia production; and waste treatment for inorganic waste from propylene oxide production).

The model shows that the total cradle-to-gate GHG emissions of the global chemical industry will reach 2.3 billion tons of CO_2_-equivalent (2.3 Gt CO_2_-eq.) per annum by 2030 (Fig. 1B). Over half of these emissions arise from the sum of NH_3_ manufacture (21%) plus the partial oxidation of hydrocarbons to oxygenates (30%). These processes should therefore receive directed efforts from the scientific community to develop alternative production processes with reduced carbon footprint. The remainder arise from the production of polyolefins (28%), aromatics (12%), chlorine (3%), vinyl chloride (3%), and acrylonitrile (2%).

We performed a further breakdown of the annual cradle-to-gate emissions and identified the major emission components to be the production of H_2_ feedstock for NH_3_ manufacture, over-oxidation of hydrocarbons to CO_2_, and fossil-based heat and electricity supply required to reach thermocatalytic process conditions (Fig. 1C and Table S3). For NH_3_ manufacture, the bulk of associated GHG emissions are direct emissions from the steam reforming of methane with subsequent water-gas-shift reactions to produce the H_2_ feedstock, which is then converted with nitrogen (N_2_) to NH_3_ via the Haber-Bosch reaction. This process accounts for 0.4 Gt CO_2_-eq. or 18% of the global chemical industry emissions (Fig. 1C).

The direct emissions of hydrocarbon oxidation processes result from limited selectivity towards the target oxygenate products, i.e., a portion of hydrocarbons oxidize all the way to CO_2_. This accounts for 0.19 Gt CO_2_-eq. (8.4% of global chemical industry emissions, Fig. 1C) and arises primarily from methanol, ethylene glycol, and terephthalic acid production. These direct emissions can be eliminated if alternative anodic hydrocarbon-to-oxygenate conversion methods are developed with near-unity selectivity. As less hydrocarbon feedstock will be required to produce a given amount of oxygenate, this will concurrently reduce feedstock emissions, i.e., emissions arising from hydrocarbon generation via naphtha cracking, which account for 2.6% of global chemical industry emissions.

To reach the required temperatures and pressures for thermocatalytic reactions, feedstocks need to be heated and compressed. For instance, present-day thermocatalytic reactions run at 200 to 300 °C for ethylene oxide, 800 °C for ammonia production, and 175 to 225 °C in the case of terephthalic acid. The heat and electrical energy come from fossil fuels today and account for 0.25 and 0.11 Gt CO_2_-eq. (Fig. 1C, i.e., 11 and 5% of global GHG emissions associated with the chemical industry). Although heat can be supplied by renewable-electricity-powered resistive heating, i.e., by retrofitting existing heating devices with electrode boilers, such electrical power-to-heat processes require vast amounts of electricity. The development of cathodic HER and anodic hydrocarbon oxidations that can be conducted at ambient temperatures and pressures will reduce the GHG emissions associated with compression and heating.

### GHG reduction potential of electrified hydrocarbon-to-oxygenates

**Fig. 2:**
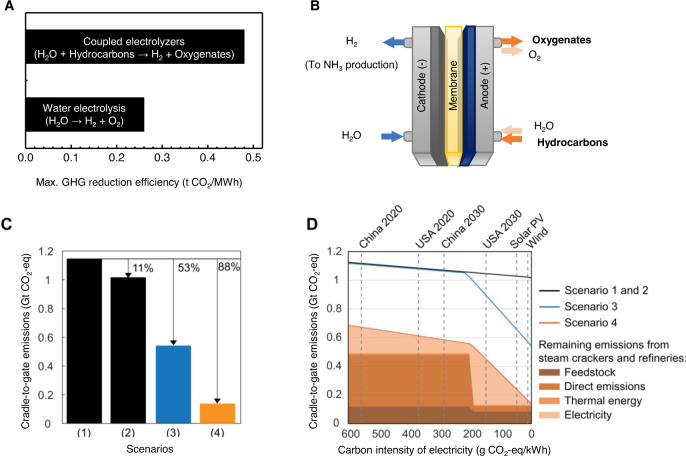
Greenhouse gas reduction potential of anodic hydrocarbon-to-oxygenate conversions. **A** Maximum greenhouse gas reduction efficiencies per MWh electricity of water electrolysis and coupled electrolyzer technologies. **B** An electrolyzer system that couples cathodic hydrogen evolution reaction with anodic hydrocarbon-to-oxygenate conversion under ambient conditions. **C** Annual cradle-to-gate emissions of NH_3_ manufacture and hydrocarbon-to-oxygenate conversions in four scenarios: (1) continuing to use fossil-based technologies, (2) using electricity only from renewable sources, without further changes to the manufacturing processes, (3) changing H_2_ production over to renewable-energy-powered water electrolysis, and (4) the inclusion of renewable-energy-powered coupled electrolyzer technologies. Note that the black line represents Scenarios 1 and 2. Scenario 1 represents the greenhouse gas emission for an electricity impact of ~750 g CO_2_-eq. per kWh and Scenario 2 the greenhouse gas emissions for an electricity impact of 0 g CO_2_-eq. per kWh. **D** Annual cradle-to-gate emissions of the scenarios considered herein vs. electricity supply having different carbon intensities.

As the bulk of GHG emissions associated with NH_3_ manufacture arise from the production of H_2_ feedstock, these emissions can be eliminated by using H_2_ produced from renewable-electricity-powered water electrolysis^14^. However, the GHG reduction efficiency of today’s water electrolysis (WE) replacing steam-methane reforming is 0.26 t CO_2_-eq. per MWh of electricity (Fig. 2A). Thus, the limited renewable electricity resource should only be allocated to water electrolysis if such more efficient options are not available.

The proposed renewable-energy-powered electrolyzer systems that couple cathodic HER with anodic hydrocarbon-to-oxygenate conversion under ambient conditions (Fig. 2B) can increase the GHG reduction efficiency to as much as 0.48 t CO_2_-eq. per MWh of electricity under ideal conditions (Fig. 2A).

We proceed to evaluate the overall GHG reduction potential of renewable-energy-powered electrolyzer systems that couple cathodic HER with anodic hydrocarbon-to-oxygenate conversion under ambient conditions (known herein as Scenario 4, Fig. 2C). The models are then used to contrast three scenarios: (1) fossil-based NH_3_ manufacture and partial oxidations of hydrocarbons to oxygenates with the current global electricity supply in ecoinvent^15^ for 2018 (~750 g CO_2_-eq. per kWh of electricity), (2) using electricity only from renewable sources, without further changes to the manufacturing processes, and (3) funneling all H_2_ production to renewable-energy-powered water electrolysis. For this purpose, we use the same LCA model as in the previous section but limit the scope to the final demand for NH_3_ and oxygenates for the year 2030 (see Supplementary Material “Final demands”). In Scenario 3, hydrogen is produced by water electrolysis. In Scenario 4, coupled electrolyzer systems are added to produce oxygenates (see Supplementary Materials “Modelling of coupled anodic hydrocarbon-to-oxygenates and cathodic hydrogen evolution reactions”). As the global demand of hydrogen for NH_3_ manufacture exceeds the demand of oxygenates, the remaining hydrogen demand unmet by the coupled electrolyzer systems is fulfilled by water electrolysis in Scenario 4. To first establish the maximum potential for GHG reductions, the renewable energy is assumed to be of zero-carbon intensity and the electrochemical routes of ideal efficiencies. With fossil-based Scenario 1 as the benchmark, Scenario 2 can reduce GHG emissions by 11%, while Scenario 3 can enable further reductions up to 53% by using hydrogen (Fig. 2C). Scenario 4 can reduce GHG emissions even further by up to 88%. The remaining 12% of emissions are due to the operation of steam crackers and refineries to provide hydrocarbon feedstock, and can be eliminated by replacing petrochemicals with cleaner and more sustainable feedstocks. This means that the coupled electrolyzer systems can avoid lock-in fossil technology by allowing for the transition from current fossil feedstocks to a completely carbon-neutral situation with green feedstocks.

In addition, we calculate the GHG reduction potential of each scenario with electricity supply of different carbon intensities (Fig. 2D). Our optimization approach minimizes the GHG emissions for the entire supply chain of the chemical industry. We note that Scenario 3 can only reduce GHG emissions with an electricity supply of low carbon intensity (i.e., <200 g CO_2_-eq. per kWh), which is not available in most industrialized countries today or even in 2030. For Scenario 4, the GHG emissions decline non-linearly; below 200 g CO_2_-eq. per kWh, the supply chain of NH_3_ manufacture switches fully from fossil- to renewable-electricity-based hydrogen production. Thus, the supply chain in Scenario 4 is based on the combination of hydrocarbon-to-oxygenate reactions and water electrolysis to produce hydrogen. More importantly, the optimization approach further determines that coupled electrolyzer technologies have the potential to reduce GHG emissions irrespective of the carbon intensity of electricity supply. GHG emissions can be reduced by up to 39% even with today’s electricity supply in the United States or China (Fig. 2D). This highlights the GHG reduction potential and the need for further research on efficient anodic hydrocarbon-to-oxygenate conversions that can be coupled to HER.

To seek insight into the operating costs of NH_3_ manufacture and hydrocarbon-to-oxygenate conversions, we estimate the annual electricity and oil-equivalent consumption in the four scenarios (Table S5). It can be seen that while the coupled electrolyzer technologies lead to an increase in annual electricity consumption for Scenario 4, the annual oil-equivalent consumption of fossil resource for feedstock and energy input decreases as well. Given that oil prices range between $50 to 71 per barrel and electricity prices range between 2 to 6 cents/kWh, this overall leads to comparable or even slightly lower energy and feedstock costs compared to Scenarios 1-3 (Fig. S7). This is true even with low oil cost (i.e., oil price at the lower bound of the range) and high electricity cost (i.e., electricity price at the upper bound of the range). This suggests benefit from investing in research and development of coupled electrolyzer technologies. While oil prices are known to fluctuate, the present model adheres to guidelines by the IEA; if oil prices increase relative to these values, the finding of comparable or even slightly lower energy and feedstock costs in Scenario 4 compared to Scenario 1–3 will be further strengthened.

An analysis of state-of-art databases shows that 18% of global chemical sites currently conduct both NH_3_ manufacture and partial oxidations of hydrocarbons to oxygenates^13^. These sites can be targeted for implementation of coupled electrolyzer technologies in the short term. These co-production sites tend to be large production sites such as the one located in Ludwigshafen, Germany. In this “Verbund”-site, almost 1 Mt of ammonia and oxygenates (i.e., methanol, ethylene oxide, and propylene oxide) are produced jointly and thus offer excellent conditions for the implementation of the coupled electrolyzer technologies.

### Industrial needs vs. present-day electrochemical technologies

**Fig. 3:**
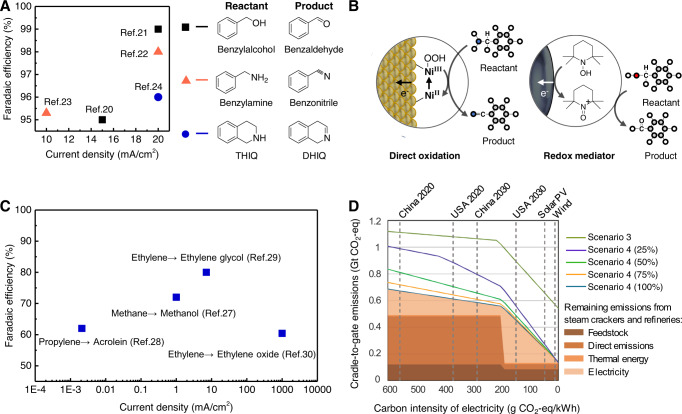
State-of-art anodic oxidation reactions. **A** Current densities and Faradaic efficiencies of reported state-of-the-art anodic oxidation reactions. **B** Direct oxidation at the anode versus redox mediator. **C** Comparison of Faradaic efficiency and current density of recent reported anodic hydrocarbon oxidations. **D** Sensitivity analysis of annual cradle-to-gate emissions with coupled electrolyzer technology of different energy efficiencies, based on Scenario 4 with electricity supply of different carbon intensities. The cradle-to-gate system boundary includes the process steps from the extraction of resources (oil, gas, renewable resources) to the production of the chemicals in scope. This assessment thus also includes consumption of power.

Having established the GHG reduction potential of the coupled electrolyzer technologies, we proceed to discuss the gap between industrial needs and state-of-art reactions. The development of anodic oxidations as a synthetic toolkit for chemical upgrading has been recently gaining attention as a means to overcome the thermodynamic limits of OER and increase the efficiency of renewable electricity conversion to chemical value. These include the anodic oxidation of alcohols to aldehydes^16,17^, amines to nitriles^18,19^, and tetrahydroisoquinolines (THIQs) to dihydroisoquinolines (DHIQs)^20^, which are thermodynamically more favorable than OER (Fig. 3A). Redox mediators can also be used to target certain functional groups and promote partial oxidation at lower applied potentials than direct oxidation at the anode^21^ (Fig. 3B). For example, the redox mediator (2,2,6,6-Tetramethylpiperidin-1-yl)oxyl (TEMPO) is often used to target alcohol groups selectively for oxidation^22^.

Target products have been achieved with high Faradaic efficiencies (>90%) (Fig. 3A); however, research to date was not focused on the primary chemicals contributing to global GHG emissions, nor the associated markets commensurable with that of NH_3_. In contrast, the electrification of hydrocarbon-to-oxygenate conversions can make a significant impact in reducing the carbon footprint of the chemicals industry, due to the high production volumes and GHG emissions. Recently, there has been progress in anodic hydrocarbon oxidations, via sp3 C-H functionalization in the case of methane^23^, in the allylic carbon of propylene^24^, and addition across the C=C bond such as in ethylene and propylene. These reactions were characterized by high Faradaic efficiencies and specificities of over 50% (Fig. 3C). High stabilities of 100 h have been achieved for C=C additions such as the dihydroxylation of ethylene and propylene^25^. However, the current densities of such reactions are generally below that required for commercial implementation (i.e., <10 mA/cm^2^). Taking the anodic production of ethylene oxide and propylene oxide for example, technoeconomic analysis showed that high current densities (i.e., 300–1000 mA/cm^2^) are required to minimize the surface area of electrochemical reactors and thereby lower capital costs^26,27^.

High energy efficiencies indicate the yield of desired chemical products for a given electrical input, particularly that of limited renewable electricity. Energy efficiency is defined as the ratio between the useful output of the electrolyzer system and the electricity input, and is related to parameters such as conversion, Faradaic efficiency, and the voltage required to drive a given current density (see Equations 8–10 in Supplementary Materials). To quantify the energy efficiencies required to meet the carbon reduction target, we perform a sensitivity analysis based on Scenario 4 with electricity of different carbon intensities (Fig. 3D). As mentioned in the previous section, Scenario 4 can enable a maximum GHG reduction potential of 88% (Fig. 2C) with a GHG reduction efficiency of 0.46 t CO_2_ per MWh of renewable electricity (Table S4). Although the GHG reduction potential of Scenario 4 decreases with decreasing energy efficiency, it is insensitive to energy efficiency if electricity can become entirely renewable (i.e., carbon intensity of 0 g CO_2_ per kWh). At 25% energy efficiency, the GHG reduction efficiency is 0.33 t CO_2_ per MWh of renewable electricity, still >25% higher than for water electrolysis at 0.26 t CO_2_ per MWh.

Lastly, we note that the anodic partial oxidation of higher carbon reactants, such as the oxidation of benzene to phenol and xylene to terephthalic acid, are also important towards mitigating GHG emissions from the chemicals industry. These reactions are less explored and should receive dedicated efforts from the scientific community.

### How should we move towards these new technologies?

To accelerate the realization of the coupled electrolyzer technology from Scenario 4, we highlight technological targets that must be achieved by researchers who wish to embark on this burgeoning research direction. An important technological target is the activation of saturated C-H bonds without the need for thermal input, in order to enable the functionalization of hydrocarbons such as methane. A potential solution is the adaptation of the Shilov system, in which the partial oxidation of a hydrocarbon such as methane to methanol or methyl chloride is catalyzed by an aqueous Pt^II^ salt with Pt^IV^ as a stoichiometric oxidant, in an electrochemical setting in which the anode acts as the oxidant^23^.

**Fig. 4:**
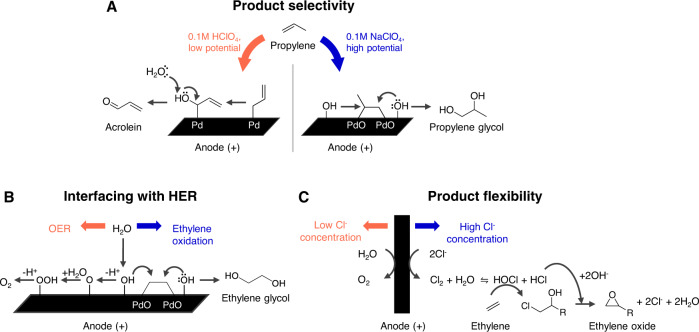
Technical targets that must be achieved for anodic hydrocarbon oxidations and reported strategies to overcome them. **A** Funnelling selectivity towards the desired product (adapted from ref. ^24^, copyright 2019 Royal Society of Chemistry). **B** The interfacing of anodic partial hydrocarbon oxidation with cathodic hydrogen evolution reaction^25^. **C** Flexibility between anodic hydrocarbon-to-oxygenates conversion and oxygen evolution reaction (adapted from ref. ^26^, copyright 2020 American Association for the Advancement of Science).

In the case of longer chain hydrocarbons, it can be challenging to selectively oxidize the desired C atom. In the case of propylene, two potential sites for partial oxidation exist (Fig. 4A); the allylic carbon, which would produce acrolein, and the unsaturated double bond, which would yield propylene oxide or glycol. Recently, the anodic oxidation of propylene to acrolein over a nanostructured palladium anode in acidic electrolyte conditions at potentials of 0.9 V vs RHE was reported at 62% Faradaic efficiency (Fig. 4A)^24^. The dihydroxylation of unsaturated C=C bonds appears to be favored over an activated palladium anode under neutral conditions and operating at relatively higher potential of 1.7 V vs RHE, with 81% Faradaic efficiency at 7.1 mA/cm^2^ towards ethylene glycol and 78% at 5.6 mA/cm^2^ towards propylene glycol. The activation of the palladium, to an oxidized form during the first two hours, increases the current density by ~3 fold and Faradaic efficiency by ~8 fold.

Another important technological target is to interface anodic partial hydrocarbon oxidation with cathodic HER; yet these operate optimally under different conditions of pH, electrolyte, operating potential. For instance, HER is conducted in aqueous conditions, which will limit the mass transport of hydrocarbon to the anode catalyst and introduce competing OER. The large positive potentials necessary for conducting these reactions at high current densities can lead to uncontrolled over-oxidation and generate undesired byproducts such as CO_2_. Control of operating potentials and electrolyte conditions is used to suppress OER and funnel Faradaic efficiency towards the desired product (Fig. 4B). Redox mediators such as Cl^−^ can also be employed at the anode with extended heterogeneous:homogeneous interfaces. The Cl^−^ buffers ethylene from uncontrolled oxidation and thereby facilitates ethylene oxide production^26^.

Since the global demand for hydrogen exceeds the demand for oxygenates, flexibility between anodic hydrocarbon-to-oxygenates conversion and OER is another important target to achieving Scenario 4 (Fig. 4C). For example, in the anodic oxidation of ethylene to ethylene oxide, the Faradaic efficiencies of ethylene oxide and O_2_ can vary depending on the concentration of Cl^−^ in the electrolyte^26^. Thus, there is the potential for flexibility to tune the hydrogen-to-oxygenate ratio as market demand evolves.

## Discussion

The anodic oxidation of hydrocarbons to oxygenates such as propylene oxide, phenol, ethylene glycol, terephthalic acid, and methanol is a synthetic toolkit with significant GHG reduction potential. However, current performance remains below that needed for commercial implementation^26^. Dedicated efforts are needed to address the associated technical challenges to electrify the chemical industry. One must bear in mind that the increased demand for renewable electricity will lead to higher demand for land and mineral resources. The land required for photovoltaics and hydropower plants is 10–20 m^2^/MWh^28,29^. Renewable technologies can also be material- and mineral-intensive; in particular, solar, wind, and electrolyzer technologies increase demand for silicon, copper, cobalt, nickel, zinc, chromium, manganese, molybdenum, and rare earth metals^30^.

It must also be noted that such electrochemical reactions are only one element of the transition towards carbon-neutrality; they do not address 12% of the cradle-to-gate emissions associated with NH_3_ manufacture and partial hydrocarbon oxidations (Fig. 2B, C), that are due to the operation of steam crackers and refineries to provide hydrocarbon feedstock such as ethylene and p-xylene. These emissions can be reduced by the substitution of thermal energy with electrified steam cracker furnaces^31^.

In a more distant future, these feedstock emissions can be eliminated by replacing petrochemical feedstocks with those from cleaner and more sustainable sources. Hydrocarbon feedstocks such as methanol, olefins, and aromatics, when produced through the electrochemical reduction of CO_2_, have the potential to close the carbon cycle^9,32^. In this way, the coupled electrolyzer systems can avoid lock-in in fossil technologies. A successful demonstration has been conducted in which CO_2_-derived ethylene is anodically converted to ethylene oxide, thereby realizing an all electrochemical route with CO_2_, water, and electricity as the only consumables^26^.

Biomass has the potential to serve not only as energy source, but also as feedstock for anodic upgrading into commodity chemicals. This use in commodity chemicals has the potential to maximize the value created per amount of biomass used. With regards to this, lignin, a material obtained from plant-based biomass, is the world’s largest source of naturally occurring aromatics. Anodic oxidation has been deployed to oxidize the alcohol groups in close vicinity to the β-O-4 ether bond, which constitutes 45-60% of the linkages in lignin, in order to facilitate its cleavage and release value add sub-units^33,34^.

Anodic upgrading can also be used to convert biomass-derived small molecules to plastic precursors and enable sustainable polymers. The sugar derivative hydroxymethylfurfural (HMF) can be anodically oxidized to 2,5-furandicarboxylic acid (FDCA), a potential replacement for terephthalic acid in polyethylene terephthalates (PET)^35–37^. The major byproduct in biodiesel production, glycerol, of which 0.1 ton will be generated per ton of biodiesel produced, can be anodically converted to lactic acid, the precursor to polylactic acid^6^. This occurs through the oxidation of the secondary alcohol group to form 1,3-dihydroxyacetone (DHA)^38,39^, followed by base-catalyzed dehydration and Cannizzarro rearrangement to form lactic acid^40^. The aforementioned examples show that, even with the maturation of CO_2_ utilization and biomass-processing technologies, anodic oxidations will be a central element of a fully renewable chemical industry.

## Methods

### General model description

The bottom-up model is based on the methodology of the Technology Choice Model (TCM)^41^ and a recent publication by the authors^8^. The model can be used to calculate the environmental impact of large production systems and complete supply chains for chemicals and plastics while being in-line with the ISO standards for LCA^42,43^. The TCM relies on the general calculation method of LCA^44^ and adapts this calculation method to represent a mathematical optimization problem, as shown in Eqs. 1 to 3.

1 $${Min} \,{h}_{{{{{{{\rm{CO}}}}}}}_{2}}={Q}_{{{{{{{\rm{CO}}}}}}}_{2}}{Bs}$$

2 $$s.t.\,{As}=y$$

3 $$0\le s\le c$$

where ${h}_{{{{{{{\rm{CO}}}}}}}_{2}}$ represents the accumulated greenhouse gas emissions, $A$ is the technology matrix, $y$ is the final demand, and $c$ is the potential upper bound for the scaling vector $s$. Matrix $B$ represents how technologies exchange elementary flows with the environment, i.e., consume natural resources and release emissions. Here, Eq. 1 defines the objective to minimize greenhouse gas (GHG) emissions from cradle-to-gate, while Eq. 2 specifies the final demand being fulfilled. Thus, the supply always meets the demand of the specified chemical products over the complete supply chain. For instance, if the final demand of ethylene oxide is the only specified demand, then the supply of ethylene would be exactly that required by the ethylene oxide production technologies.

The elements in ${Q}_{{{{{{{\rm{CO}}}}}}}_{2}}$ represent the 100-year global warming potential of each elementary flow according to IPCC 2014^45^. Thus, the objective function represents the accumulated equivalent CO_2_ emissions. Equation 3 defines that the scaling vector entries must be between zero and the upper bound $c$. The upper bound $c$ can be used to limit the supply of electricity or thermal energy to a specific technology. All technologies in the matrices $A$ and $B$ are described by full mass- and energy balances. The matrices of the model are provided on Zenodo^46^ whenever there are no additional licenses required. There are 429 variables (processes in *A*) and 174 constraints (flows in *y*) included in the analysis. *c* is set to infinite.

### Overview of datasets

For the model, we construct the technology matrix *A* and elementary flow matrix *B* from process data on the level of individual processes. These processes are either unit or aggregated datasets.

#### Unit process datasets

Unit process datasets are the smallest possible entity of a process with inputs and outputs. On this unit level, the dataset considers all technical inputs and outputs. Only the elementary flows of a single unit process are represented by the dataset.

#### Aggregated process datasets

Unlike unit process datasets that represent only a single unit process, fully terminated aggregated process datasets represent the entire production chain. For aggregated process datasets, elementary flows enter or exit, and only one technical flow exits the process. Other technical flows are created or consumed within the production chain.

### Dataset selection

The methodology proposed by Kätelhön et al. is used to select the respective chemical and plastic production technologies^41^. In this procedure, commercially available technologies that lead to the lowest greenhouse gas emissions, which are denoted best available technologies^2,47,48^, are first identified. The mass and energy balances for all best available technologies are then included as unit process datasets. Next, we identify relevant renewable ammonia production technologies, as well as coupled anodic hydrocarbon-to-oxygenates and cathodic hydrogen evolution reactions based on literature review. We then include the unit processes for these technologies (see the following sections for further details). Lastly, aggregated datasets are included for all missing inputs (e.g., chemicals, thermal energy, and other utilities) not provided by one of the unit processes already included in the previous steps.

A list of datasets can be found in Table S6. Part of this table has been originally published by Meys et al.^8^.

### Aggregated datasets

Aggregated datasets are from the LCI database ecoinvent^15^ and represent the global production mix (global dataset). If a global dataset is not available, a European dataset is used instead.

### Used unit-process datasets

#### State-of-the-art processes

Datasets for conventional production routes are obtained from the IHS Process Economics Program^49^. This database contains process simulations and datasets that have been verified by industrial experts. As it does not include elementary and waste flow, such as inorganic or organic residues, we adapted and included a model for waste incineration^50,51^, based on data on hazardous waste incineration in a chemical park in western Germany. In this model, flue gas cleaning is based on wet scrubbers, electrostatic precipitators, and SCR-low dust and SNCR DeNOx stages.

#### Ammonia production from hydrogen and nitrogen

NH_3_ is produced through the Haber-Bosch process, which uses a gaseous mixture of H_2_ and N_2_ as the feed to a reactor operating at 450 °C and 200 bar. After the reaction, liquid NH_3_ is cryogenically separated from the product mixture. Unconverted H_2_ and N_2_ are recycled back to the Haber-Bosch reactor^52^. Our model utilizes the unit process data from an existing scientific publication^53^. The H_2_ feed is either supplied by water electrolysis or other H_2_ production processes, while N_2_ is supplied by an air separation unit.

#### Water electrolysis

To model electrochemical H_2_ and O_2_ production, we use the process data of low-temperature water electrolysis. The average efficiency of this process is 67.15% based on net calorific values, resulting in an electricity consumption of 178.7 MJ per kg of H_2_ produced^7^.

### Modeling principles of coupled electrolyzer systems

In this publication, electrolyzer systems that couple cathodic HER with anodic hydrocarbon-to-oxygenate conversion have been added to the existing model of the chemical industry. As coupled electrolyzer technologies are still in the early development stages, unit process data for these reactions is therefore unavailable. To fill these data gaps, we assume stoichiometric reactions with full conversion for the unit processes:

4 $${v}_{{{{{{\rm{Hydrocarbon}}}}}}} \,{M}_{{{{{{\rm{Hydrocarbon}}}}}}}+{v}_{{{{{{{\rm{H}}}}}}}_{2}{{{{{\rm{O}}}}}}}{M}_{{{{{{{\rm{H}}}}}}}_{2}{{{{{\rm{O}}}}}}}\to {v}_{{{{{{\rm{Oxygenate}}}}}}}\,{M}_{{{{{{\rm{Oxygenate}}}}}}}+{v}_{{{{{{{\rm{H}}}}}}}_{2}}{M}_{{{{{{{\rm{H}}}}}}}_{2}}$$

where $v$ denotes the stoichiometric coefficient and $M$ the molar mass. The list of all considered anodic hydrocarbons-to-oxygenates conversions can be found in Table S1. The mass flow $m$ of each reactant and product $i$ can then be calculated based on the stoichiometric reaction:

5 $${m}_{i}=\frac{{v}_{i}{M}_{i}}{{v}_{{{{{{\rm{Oxygenate}}}}}}}\,{M}_{{{{{{\rm{Oxygenate}}}}}}}}$$

The electricity requirement ${P}_{{{{{{\rm{el}}}}}},{{\min }}}$ of the electrochemical reaction is equivalent to the enthalpy of reaction ${\triangle H}_{{{{{{\rm{R}}}}}}}^{0}$, which is calculated from the stoichiometric coefficient $v$ and the standard enthalpy of formation $\triangle {h}_{{{{{{\rm{f}}}}}}}^{0}$ of each reactant and product $i$ (see Table S2 for the standard enthalpy of formation of each compound):

6 $${P}_{{{{{{\rm{el}}}}}},{{\min }}}=\triangle {H}_{{{{{{\rm{R}}}}}}}^{0}=\mathop{\sum}\limits_{i}{v}_{i}\triangle {h}_{{{{{{\rm{f}}}}}},i}^{0}$$

### Final demands

The final demand is the total output or the amount of waste treatment of an intermediate flow. In this publication, two final demands are considered:

(1) the final demand for the entire chemical industry, which is used to derive the results shown in Fig. 1B, C, and

(2) the final demand for ammonia and oxygenates only, as given in Table S3 and used to calculate the results shown in Fig. 2C, D.

### Calculation of the GHG reduction efficiency

The GHG reduction efficiency of scenario $s$ is calculated as the ratio of the GHG reduction, which is obtained by subtracting the GHG emissions of scenario $s$ from that of the scenario in which fossil fuels continue to be utilized, to the electricity demand of scenario $s$:

7 $${\eta }_{{{{{{\rm{GHG\; reduction}}}}}},s}=\frac{{{{{{{\rm{GHG}}}}}}}_{{{{{{\rm{fossil}}}}}}}-{{{{{{\rm{GHG}}}}}}}_{s}}{{{{{{{\rm{W}}}}}}}_{{{{{{\rm{el}}}}}},s}}$$

This scenario-specific definition of the GHG reduction efficiency generalizes the technology-specific definition by Sternberg et al., in which the GHG emissions of a new technology is compared to that of an incumbent technology^54^. Figure 2A shows the scenario-specific GHG reduction efficiencies for water electrolysis and coupled electrolyzer systems.

## Supplementary information

Supplementary Information

## Data Availability

The model parameters as well as all data used are publicly available. However, a license by IHS Markit is required in some cases. All data that can be made openly available is provided via Zenodo (https://zenodo.org/record/5118762#.YWacOByxWUk)^46^.

## References

[1] IEA. *The Future of Petrochemicals: Towards More Sustainable Plastics and Fertilisers* (IEA, 2018).

[2] IEA. *Technology Roadmap - Energy and GHG Reductions in the Chemical Industry via Catalytic Processes* (IEA, 2013).

[3] Boulamanti, A. & Moya, J. A. *Energy Efficiency and GHG Emissions: Prospective Scenarios for the Chemical and Petrochemical Industry* (2017).

[4] Ogden JM (1999). Prospects for building a hydrogen energy infrastructure. Annu. Rev. Environ. Resour..

[5] Birol, F. *The Future of Hydrogen: Seizing Today’s Opportunities* (IEA, 2019).

[6] Verma S, Lu S, Kenis PJA (2019). Co-electrolysis of CO_2_ and glycerol as a pathway to carbon chemicals with improved technoeconomics due to low electricity consumption. Nat. Energy.

[7] Agora, E. *The Future Cost of Electricity-Based Synthetic Fuels*. Report (2018).

[8] Meys R (2021). Achieving net-zero greenhouse gas emission plastics by a circular carbon economy. Science.

[9] Kätelhön A, Meys R, Deutz S, Suh S, Bardow A (2019). Climate change mitigation potential of carbon capture and utilization in the chemical industry. Proc. Natl Acad. Sci. USA.

[10] Gabrielli P, Gazzani M, Mazzotti M (2020). The role of carbon capture and utilization, carbon capture and storage, and biomass to enable a net-zero-CO_2_ emissions chemical industry. Ind. Eng. Chem. Res..

[11] Mac Dowell N, Fennell PS, Shah N, Maitland GC (2017). The role of CO_2_ capture and utilization in mitigating climate change. Nat. Clim. Change.

[12] D’Ambrosio, D. *Tracking Power 2020* (IEA, 2020).

[13] Carbon Minds (cm.chemicals database, 2020).

[14] Bhandari R, Trudewind CA, Zapp P (2014). Life cycle assessment of hydrogen production via electrolysis—a review. J. Clean. Prod..

[15] ecoinvent. ecoinvent Data V. 3.5 (Swiss Centre for Life Cycle Inventories, 2019).

[16] Zheng J (2017). Hierarchical porous NC@CuCo nitride nanosheet networks: highly efficient bifunctional electrocatalyst for overall water splitting and selective electrooxidation of benzyl alcohol. Adv. Funct. Mater..

[17] Chen X (2019). Defect engineering of nickel hydroxide nanosheets by Ostwald ripening for enhanced selective electrocatalytic alcohol oxidation. Green. Chem..

[18] Huang Y, Chong X, Liu C, Liang Y, Zhang B (2018). Boosting hydrogen production by anodic oxidation of primary amines over a NiSe nanorod electrode. Angew. Chem. Int. Ed..

[19] Ding Y (2020). Benzylamine oxidation boosted electrochemical water-splitting: Hydrogen and benzonitrile co-production at ultra-thin Ni_2_P nanomeshes grown on nickel foam. Appl. Catal. B Environ..

[20] Huang C, Huang Y, Liu C, Yu Y, Zhang B (2019). Integrating hydrogen production with aqueous selective semi-dehydrogenation of tetrahydroisoquinolines over a Ni_2_P bifunctional electrode. Angew. Chem. Int. Ed..

[21] Reid LM, Li T, Cao Y, Berlinguette CP (2018). Organic chemistry at anodes and photoanodes. Sustain. Energ. Fuels..

[22] Rafiee M, Miles KC, Stahl SS (2015). Electrocatalytic alcohol oxidation with TEMPO and bicyclic nitroxyl derivatives: driving force trumps steric effects. J. Am. Chem. Soc..

[23] Kim RS, Surendranath Y (2019). Electrochemical reoxidation enables continuous methane-to-methanol catalysis with aqueous Pt salts. ACS Cent. Sci..

[24] Winiwarter A (2019). Towards an atomistic understanding of electrocatalytic partial hydrocarbon oxidation: propene on palladium. Energy Environ. Sci..

[25] Lum Y (2020). Tuning OH binding energy enables selective electrochemical oxidation of ethylene to ethylene glycol. Nat. Catal..

[26] Leow WR (2020). Chloride-mediated selective electrosynthesis of ethylene and propylene oxides at high current density. Science.

[27] Li Y (2022). Redox-mediated electrosynthesis of ethylene oxide from CO_2_ and water. Nat. Catal..

[28] UNECE. *Lifecycle Assessment of Electricity Generation Options* (United Nations Economic Commission for Europe, 2021).

[29] Ritchie, H. *How Does the Land Use of Different Electricity Sources Compare?* (2022).

[30] IEA. *The Role of Critical Minerals in Clean Energy Transitions*. https://www.iea.org/reports/the-role-of-critical-minerals-in-clean-energy-transitions (2021).

[31] Wismann ST (2019). Electrified methane reforming: a compact approach to greener industrial hydrogen production. Science.

[32] Barecka, M. H., Ager, J. W. & Lapkin, A. A. Economically viable CO_2_ electroreduction embedded within ethylene oxide manufacturing. *Energy Environ. Sci*. **14**, 1530–1543 (2021).

[33] Rafiee M, Alherech M, Karlen SD, Stahl SS (2019). Electrochemical aminoxyl-mediated oxidation of primary alcohols in lignin to carboxylic acids: polymer modification and depolymerization. J. Am. Chem. Soc..

[34] Bosque I, Magallanes G, Rigoulet M, Kärkäs MD, Stephenson CRJ (2017). Redox catalysis facilitates lignin depolymerization. ACS Cent. Sci..

[35] Cha HG, Choi K-S (2015). Combined biomass valorization and hydrogen production in a photoelectrochemical cell. Nat. Chem..

[36] Jiang N, You B, Boonstra R, Terrero Rodriguez IM, Sun Y (2016). Integrating electrocatalytic 5-hydroxymethylfurfural oxidation and hydrogen production via Co–P-derived electrocatalysts. ACS Energy Lett..

[37] You B, Jiang N, Liu X, Sun Y (2016). Simultaneous H_2_ generation and biomass upgrading in water by an efficient noble-metal-free bifunctional electrocatalyst. Angew. Chem. Int. Ed..

[38] Liu D (2019). Selective photoelectrochemical oxidation of glycerol to high value-added dihydroxyacetone. Nat. Commun..

[39] Kwon Y, Birdja Y, Spanos I, Rodriguez P, Koper MTM (2012). Highly selective electro-oxidation of glycerol to dihydroxyacetone on platinum in the presence of bismuth. ACS Catal..

[40] Dai C (2017). Electrochemical production of lactic acid from glycerol oxidation catalyzed by AuPt nanoparticles. J. Catal..

[41] Kätelhön A, Bardow A, Suh S (2016). Stochastic technology choice model for consequential life cycle assessment. Environ. Sci. Technol..

[42] ISO 14040.* Environmental Management—Life Cycle Assessment—Principles and Framework* (2006).

[43] ISO 14044. *Environmental Management—Life Cycle Assessment—Requirements and Guidelines* (2006).

[44] Heijungs, R. & Suh, S. *The Computational Structure of Life Cycle Assessment* Vol. 11 (Springer, 2002).

[45] IPCC. *Climate change 2013: The physical science basis. Contribution of Working Group I to the Fifth Assessment Report of the Intergovernmental Panel on Climate Change*. eds. T. F. Stocker et al., (Cambridge Univ. Press, 2013).

[46] Meys, R. Bene94/Plastic-supply-chain-model-including-matlab-code-: V1.0. 10.5281/zenodo.5118762 (2021).

[47] Falcke, H. et al. *Best available techniques (BAT) reference document for the production of large volume organic chemicals* (EUR 28882 EN, Publications Office of the European Union, 2017).

[48] European Commission, Joint Research Centre. *Best available techniques (BAT) reference document for the production of polymers* (Publications Office of the European Union, 2007).

[49] Markit, I. H. S. *Process Economics Program (PEP) Yearbook* (2018).

[50] Doka, G. *Life Cycle Inventories of Waste Treatment Services* (Dübendorf, 2003).

[51] Doka, G. *Updates to Life Cycle Inventories of Waste Treatment Services—Part II: Waste Incineration* (Zurich, 2013).

[52] Elvers, B. & Ullmann, F. *Ullmann’s Encyclopedia of Industrial Chemistry* 7 (Wiley-VCH, 2011).

[53] Cesaro Z, Ives M, Nayak-Luke R, Mason M, Bañares-Alcántara R (2021). Ammonia to power: forecasting the levelized cost of electricity from green ammonia in large-scale power plants. Appl. Energy.

[54] Sternberg A, Bardow A (2015). Power-to-what?—environmental assessment of energy storage systems. Energy Environ. Sci..

[55] Wernet G (2016). The ecoinvent database version 3 (part I): overview and methodology. Int. J. Life Cycle Assess..

[56] Matzen, M. J., Alhajji, M. H. & Demirel, Y. Technoeconomics and Sustainability of Renewable Methanol and Ammonia Productions Using Wind Power-based Hydrogen. 10.4172/2090-4568.1000128 (2015).

[57] IEA. *Energy Technology Perspectives 2020*. https://www.iea.org/reports/energy-technology-perspectives-2020 (2020).

