# Supplementary material for: Electrified hydrocarbon-to-oxygenates coupled to hydrogen evolution for efficient greenhouse gas mitigation
Source: Nat Commun. 2023 Apr 7;14:1954. doi: 10.1038/s41467-023-37382-3 (PMC10082166; doi:10.1038/s41467-023-37382-3)
Supplement: Supplementary file 1 — Supplementary Information [file 41467_2023_37382_MOESM1_ESM.pdf]

Electrified hydrocarbon-to-oxygenates coupled to hydrogen evolution for efficient greenhouse gas mitigation

Wan Ru Leow,[1,2,†,*] Simon Völker,[3,†] Raoul Meys,[3,4,†] Jianan Erick Huang,[1] Shaffiq A. Jaffer,[5] André Bardow,[3,6,7,*] Edward H. Sargent[1,*]

[1]Department of Electrical and Computer Engineering, University of Toronto; 10 King's College Road, Toronto, Ontario, M5S 3G4, Canada.

[2]Institute of Sustainability for Chemicals, Energy and Environment (ISCE[2]), Agency for Science, Technology and Research (A*STAR); 1 Pesek Road, Jurong Island, Singapore 627833, Singapore.

[3]Institute of Technical Thermodynamics, RWTH Aachen University; Schinkelstr. 8, 52062 Aachen, Germany.

[4]Carbon Minds GmbH; Eupener Straße 165, 50933 Cologne, Germany.

[5]TOTAL American Services Inc.; Hopkinton, MA 01748, USA.

[6]Energy & Process Systems Engineering, Department of Mechanical and Process Engineering, ETH Zürich; 8092 Zürich, Switzerland.

[7]Institute of Energy and Climate Research - Energy Systems Engineering (IEK-10); Forschungszentrum Jülich GmbH, 52425 Jülich, Germany

*Corresponding authors. Email: wanru_leow@isce2.a-star.edu.sg; abardow@ethz.ch; ted.sargent@utoronto.ca

†These authors contributed equally to this work.

Supplementary Figures

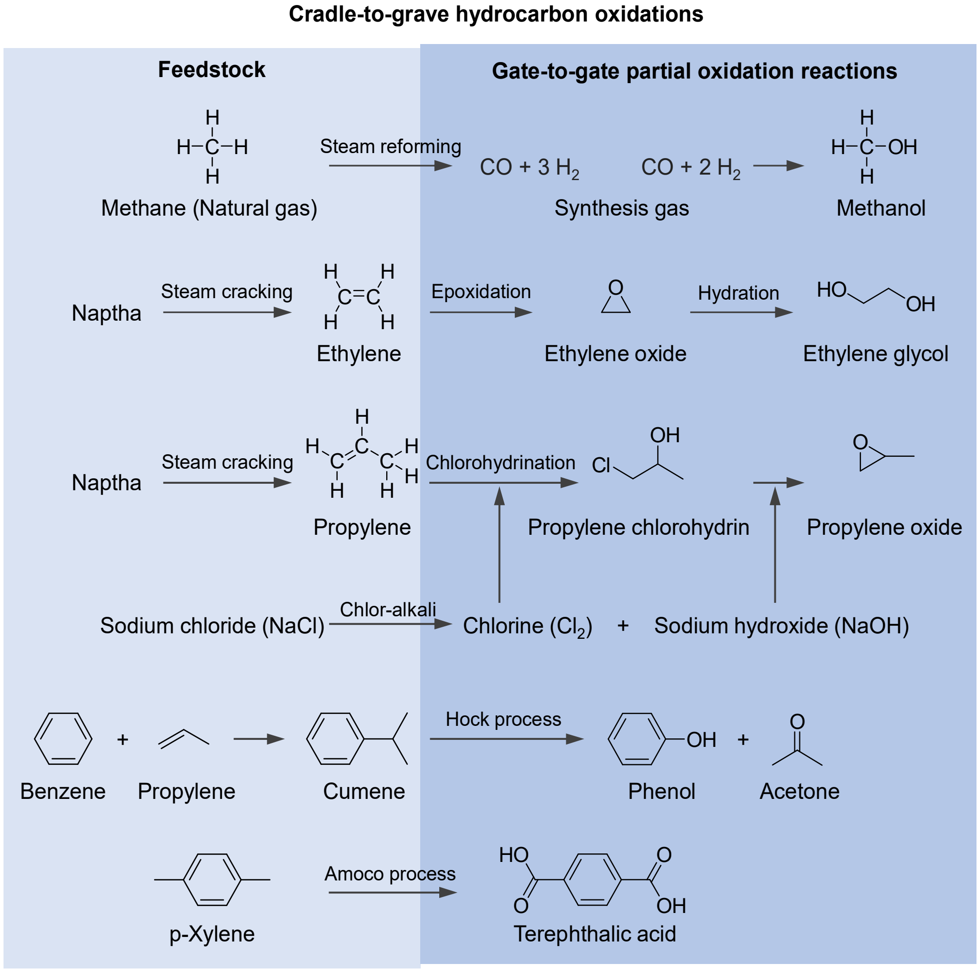

Supplementary Figure 1 Cradle-to-gate hydrocarbons-to-oxygenates processes.

Supplementary Note 1: Methanol

Currently, methanol is mostly produced via the catalytic conversion of synthesis gas. In a first step, the synthesis gas is produced by steam reforming. In the steam reforming process, the natural gas reacts over a catalyst in the absence of oxygen, while the partial oxidation process takes place without a catalyst in the presence of oxygen (second reformer). In the next step, the obtained syngas with a composition close to 2:1 is catalytically synthesized to methanol. The synthesis reaction is carried out in multiple passes, as only around 50% of the synthesis gas can be converted to methanol in one pass. Thus, the obtained methanol and co-product water is condensed out of the system, while the remaining synthesis gas is compressed and recycled to the synthesis reactor. In a low-pressure reactor, the synthesis of methanol takes place at around 200-300°C and 5-10 MPa. Finally, the obtained methanol is separated from water and other light and heavy impurities using a two-stage distillation column.[1]

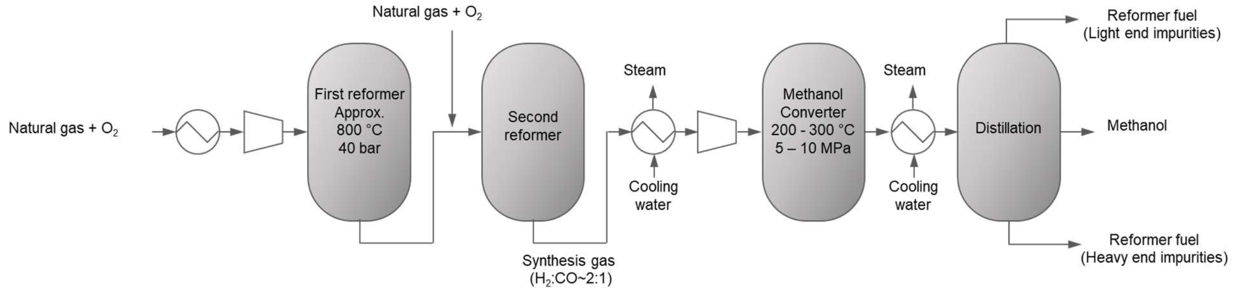

Supplementary Figure 2 Conventional process of manufacturing methanol from natural gas.

Supplementary Note 2: Terephthalic acid

Terephthalic acid is one main precursor for polyethylene terephthalate (PET) production. Terephthalic acid is produced by the partial oxidation of p-xylene in acetic acid over a catalyst. The catalyst used is composed of manganese, bromine, and cobalt. The oxidation step occurs at 175-225° C and 15-30 bar. The oxidation output flow undergoes purification. During purification, the catalyst and the acetic acid are recovered, and impurities such as 4-formylbenzoic acid are converted into p-toluic and benzoic acid. The conversion of 4-formylbenzoic acid occurs by hydrogenation at temperatures higher than 260 °C. In addition, further process steps such as crystallization, filtration, and drying are required to generate pure terephthalic acid.[1]

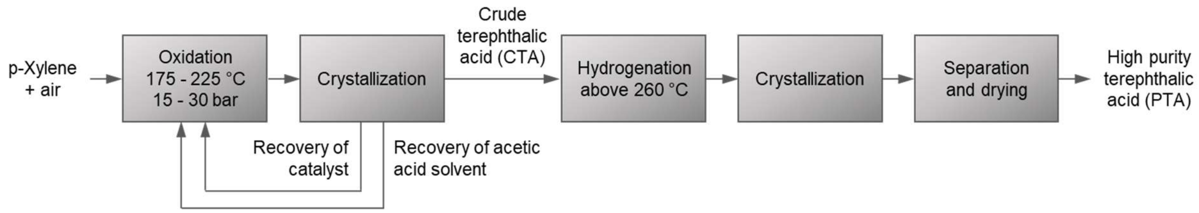

Supplementary Figure 3 Conventional process of manufacturing terephthalic acid from p-xylene.

Supplementary Note 3: Ethylene oxide and ethylene glycol
The direct oxidation of ethylene produces ethylene oxide with air or pure oxygen. The current main process technology applied is the pure oxygen-based process. The oxygen-based process takes place at 200 - 300°C and 10 - 30 bar. Water, heat, and flue gas containing carbon dioxide are co-produced during the oxidation step. While the heat is recovered, the flue gas containing the carbon dioxide is scrubbed to obtain carbon dioxide as a by-product. The scrubbed flue gas is recycled back to the oxidation step. The co-produced water leaves the oxidation step within the ethylene oxide solution. This aqueous ethylene oxide solution is first stripped and second distillated to separate water and gain purified ethylene oxide.[1]

The ethylene oxide is hydrolyzed to produce ethylene glycol at approx. 200 °C and 35 bar. About 90 % of the ethylene oxide can be converted into mono-ethylene glycol (MEG). During the hydrolysis process, heavy glycols are produced as by-products. First, the remaining water in the product mixture is removed. Second, the product mixture passes successive distillation columns to yield purified mono-ethylene glycol.[1]

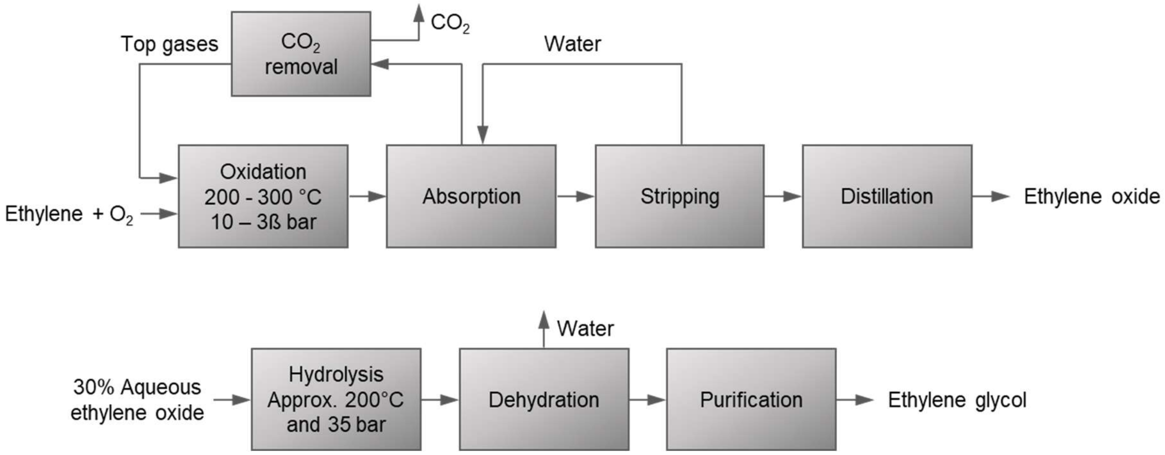

Supplementary Figure 4 Conventional process of manufacturing ethylene glycol from ethylene.

Supplementary Note 4: Phenol and acetone

Most of the phenol and acetone is produced via the cumene process, also referred to as Hock process. In the Hock process, the raw material cumene is oxidized in air in an oxidation reactor at a pressure of around 700 kPa and 80-120°C. During the oxidation step, a cumene radical is formed and reacted with oxygen to produce another cumene radical and cumene hydroperoxide, separated in the separation step. The cumene radical feeds back to the oxidation reactor while the cumene hydroperoxide undergoes a hydrolysis reaction. The hydrolysis takes place in a strong mineral acid, often sulfuric acid, to produce phenol and acetone. After distillation, the byproduct alpha-methylstyrene is hydrogenated back to cumene and recycled to the oxidation step.

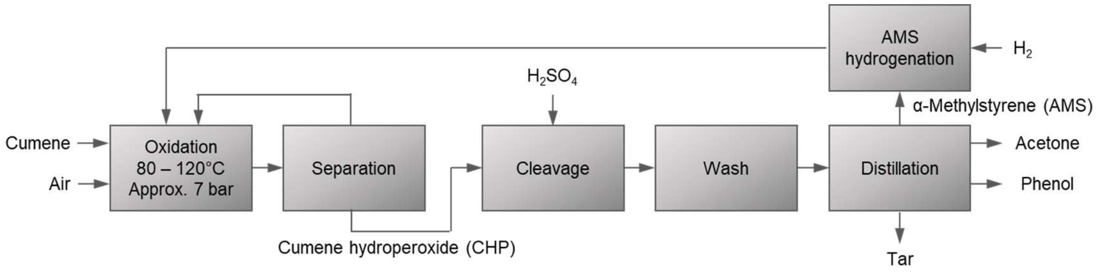

Supplementary Figure 5 Conventional process of manufacturing acetone and phenol from cumene.

Supplementary Note 5: Propylene oxide

Propylene oxide is produced from propylene and chlorine via the chlorohydrin route. In the first chlorohydrin step, propylene and chlorine react at temperatures between 45° C and 90° C at around 1.5 bar to propylene chlorohydrin and the main byproduct propylene dichloride. Propylene chlorohydrin and the other reaction products are separated and propylene chlorohydrin is feed to a saponifier and the other substances to a scrubber. In the saponifier, propylene chlorohydrin is mixed with lime and the dehydrochlorination from propylene chlorohydrin to propylene oxide and hydrogen chloride takes place. The final purification section contains condensation sections and distillation towers to separate first the light ends and further the heavier byproducts mainly propylene dichloride.

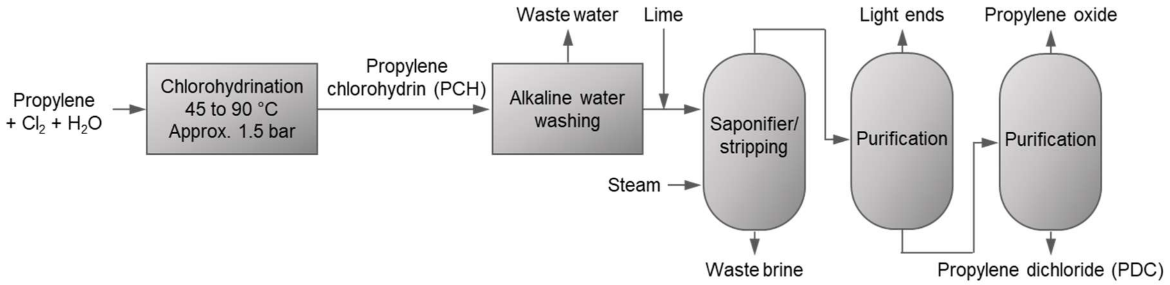

Supplementary Figure 6 Conventional process of manufacturing propylene oxide from propylene.

Supplementary Note 6: Definitions of electrochemical parameters

$$\text{Energy efficiency} = \frac{\text{Useful energy output}}{\text{Energy input}} \times 100\% \tag{8}$$

$$\text{Energy efficiency} = \text{Voltage efficiency} \times \text{Faradaic efficiency} \tag{9}$$

$$\text{Voltage efficiency} = \frac{\text{Thermodynamic potential of reaction}}{\text{Actual potential of reaction}} \times 100\% \tag{10}$$

Supplementary Table 1 Standard enthalpies of formation ΔH_R^0.

Compound	ΔH_R^0 (kJ/mol)	State	Reference[2]
Acetone	-249.4	Liquid	NIST
Benzene	49.0	Liquid	NIST
Ethylene	52.5	Gaseous	NIST
Ethylene glycol	-460.0	Liquid	NIST
Ethylene oxide	-95.7	Liquid	NIST
Hydrogen	0.0	Gaseous	
Methane	-74.9	Gaseous	NIST
Methanol	-238.4	Liquid	NIST
Phenol	-162.2	Liquid	ATcT[3]
Propylene	20.4	Gaseous	NIST
Propylene oxide	-122.6	Liquid	NIST
p-Xylene	-24.4	Liquid	NIST
Terephthalic acid	-816.3	Solid	NIST
Water	-285.8	Liquid	NIST
Water	-241.8	Gaseous	NIST

Supplementary Table 2 Final demands for the chemicals and plastics in 2030[4]. The chemicals marked with “*” are chemicals included in assessing the global climate impact of NH_3 manufacture and partial hydrocarbon oxidations. The values in brackets represent values used to assess the greenhouse gas reduction potential of anodic hydrocarbon-to-oxygenate conversions, during which the scope was limited to ammonia and oxygenates only.

Product	Final demand [Mt-a]
Acetone*	10.68 (10.68)
Acrylonitrile	10.42
Ammonia*	220.12 (229.07)
Benzene	11.06
Caprolactam	3.48
Cumene	0.91
Diethylene glycol*	4.4 (5.6)
Ethylene	10.55
Ethylene glycol*	41.66 (52.85)
Ethylene oxide*	10.45
Methanol*	135.42 (135.42)
Phenol*	17.35 (17.35)
Polyethylene	163.19
Polypropylene	100.71
Propylene	2.93
Propylene oxide*	13.89 (13.89)
Styrene	48.61
Terephthalic acid*	100.69 (100.69)
Toluene	31.29
Vinyl chloride	55.56
Xylene (mixed)	3.69
Xylene (para)	1.36

Supplementary Table 3 Tabulated values of Figure 1C of the main text.

Feedstock	Product	Feedstock (Mt CO_2-eq.)	Direct emissions and waste treatment (Mt CO_2-eq.)	Thermal energy (Mt CO_2-eq.)	Electricity (Mt CO_2-eq.)
Various feedstock	All oxygenates	130.8	188.4	253.6	105.6
Natural gas	Ammonia	45.1	402.1	0.0	22.0
Natural gas	Methanol	44.4	83.7	133.5	16.2
p-Xylene	Terephthalic acid	43.0	29.8	75.7	29.9
Ethylene	Ethylene glycol	18.3	42.5	0.0	24.0
Cumene	Phenol, acetone	15.1	19.6	20.4	4.8
Sodium chloride, Propylene	Propylene oxide	10.0	13.0	23.9	30.8

Supplementary Table 4 Tabulated values of maximum reduction potential of Scenario 4 with reduced energy efficiencies of 75%, 50%, and 25%.

All values for Scenario 4	75%	50%	25%
Power-to-X efficiency in t CO_2 per MWh for 0 g per kWh electricity impact	0.44	0.40	0.33
Maximum reduction potential	88%	88%	88%

Supplementary Table 5 Annual electricity and oil-equivalent consumption of NH_3 manufacture and hydrocarbon-to-oxygenate conversions in four scenarios: (1) continuing to use fossil-based technologies, (2) using electricity only from renewable sources, without further changes to the manufacturing processes, (3) changing H_2 production over to renewable-energy-powered water electrolysis, and (4) the inclusion of renewable-energy-powered coupled electrolyzer technologies.

Scenario	Annual electricity consumption (PWh)	Annual oil-equivalent consumption (billion barrels)
1	0.18	4.46
2	0.18	4.46
3	2.37	3.11
4	2.10	1.99

Supplementary Table 6 Summary of sources for the chemical production technologies used in the model. Part of this table has been originally published by Meys et al.[5]

Product	Technologies	Source	Comment
Acetic acid	Carbonylation of methanol	Ref. [6]	
Acetone	Oxidation of cumene	Ref. [6]	Most industrially relevant technology[1]
Acetonitrile	Market for acetonitrile	Ref. [7]	
Acrylic acid, ester grade	From propylene by ammoxidation	Ref. [6]	
Acrylonitrile	Propylene ammoxidation	Ref. [6]	
Adipic acid	Benzene oxidation via cyclohexanol	Ref. [6]	
Allyl chloride	Propylene chlorination	Ref. [6]	
Ammonia	Haber-Bosch process	Ref. [8]	
Ammonium sulfate	Market for ammonium sulfate	Ref. [7]	
Aniline	Reduction of nitrobenzene	Ref. [6]	
Benzene	Solvent extraction from pyrolysis gasoline	Ref. [6]	
Benzene	Solvent extraction from pyrolysis gasoline	Ref. [6]	
Benzene	Separation of xylenes by adsorption	Ref. [6]	
Benzene	Separation of xylenes by crystallization	Ref. [6]	
Butadiene	Market for butadiene	Ref. [7]	
1-Butene	Market for butene, mixed	Ref. [7]	
Calcium chloride	Market for calcium chloride	Ref. [7]	
Calcium oxide	Market for lime	Ref. [7]	
Caprolactam	Production from toluene	Ref. [6]	
Carbon monoxide	Partial condensation of synthesis gas	Ref. [6]	
Chlorine	Electrolysis of hydrochloric acid	Ref. [6]	
Chlorine	Electrolysis via oxygen-depolarized cath-odes	Ref. [6]	
Chlorine/ caustic soda (50%)	Electrolysis of NaCl in membrane cell	Ref. [6]	
Chlorine/ caustic soda (50%)	Electrolysis of NaCl in diaphragm cell	Ref. [6]	
Chlorine / caustic soda (50%)	Electrolysis of NaCl in mercury cell	Ref. [6]	
Cooling water	Market for water, decarbonised, at user	Ref. [7]	
Cumene	Alkylation of benzene with propylene	Ref. [6]	
Cyclohexane	Hydrogenation of benzene	Ref. [6]	
Deionized water	Market for water, decarbonised, at user	Ref. [7]	
Dimethyl terephthalate	Esterification of terephthalic acid with methanol	Ref. [6]	

Product	Technologies	Source	Comment
Dinitrotoluene	Nitration of toluene	Ref. [6]	
Epichlorohydrin	Chlorohydrination of allyl chloride	Ref. [6]	
Ethane	Ethane extraction, from natural gas liquids	Ref. [7]	
Ethylbenzene	Alkylation of benzene with ethylene (zeolite catalyst)	Ref. [6]	
Ethylene	Steam cracking of naphtha or ethane	Ref. [6]	
Ethylene glycol, diethylene glycol	Thermal hydration of ethylene oxide	Ref. [6]	
Ethylene oxide	Ethylene oxidation with oxygen	Ref. [6]	
Formaldehyde	Oxidation of methanol (ferric-molybdate catalyst)	Ref. [6]	
Formaldehyde	Oxidation of methanol (silver catalyst)	Ref. [6]	
Gasoline	Market for petrol, unleaded	Ref. [7]	
Glycerin	Oxidation of allyl chloride via epichlorohydrin	Ref. [6]	
Hexamethylenediamine	From acrylonitrile via diponitrile	Ref. [6]	
Hexamethylenediamine	From acrylonitrile via diponitrile (electrohydrodimerization)	Ref. [6]	
Hydrochloric acid	Market for hydrochloric acid, without water	Ref. [7]	
Hydrogen	Steam methane reforming and water-gas-shift	Ref. [6]	
Hydrogen	Low-temperature electrolysis	Ref. [9]	
Hydrogen cyanide	Market for hydrogen cyanide	Ref. [7]	
Methanol	From synthesis gas	Ref. [6]	
Methyl acrylate	Esterification of acrylic acid	Ref. [6]	
Methylene diphenyl diisocyanate	Phosgenation of benzene	Ref. [6]	
Naphtha	Market for naphtha	Ref. [7]	
Natural gas (raw material)	Market for natural gas, high pressure	Ref. [7]	
Nitric acid (60%)	From ammonia (dual pressure)	Ref. [6]	
Nitric acid (60%)	From ammonia (mono pressure)	Ref. [6]	
Nitrobenzene	Nitration of benzene (adiabatic)	Ref. [6]	
Nitrobenzene	Nitration of benzene (conventional)	Ref. [6]	
Nitrogen	Air separation by pressure-swing adsorption	Ref. [6]	
n-Pentane	Market for pentane	Ref. [7]	
Oleum (33.3%)	33.3% Oleum from sulfur trioxide and sulfuric acid	Own calculation	Own calculations: 0.333 kg sulfur trioxide + 0.667 kg sulfuric acid
Oxygen	Cryogenic air separation	Ref. [6]	
o-Xylene	Separation of xylenes by adsorption	Ref. [6]	

Product	Technologies	Source	Comment
o-Xylene	Separation of xylenes by crystallization	Ref. [6]	
Phenol	Oxidation of cumene	Ref. [6]	Most industrially relevant technology[1]
Polyethylene, HD	Gas-phase polymerization	Ref. [6]	
Polyethylene, LD	Autoclave polymerization	Ref. [6]	
Polyethylene, LLD	Solution polymerization	Ref. [6]	
Polypropylene	Gas-phase polymerization	Ref. [6]	
Process water	Market for water, decarbonized, at user	Ref. [7]	
Propylene	Dimerization of ethylene	Ref. [6]	
Propylene	Ethylene disproportionation	Ref. [6]	
Propylene	Steam cracking of naphtha	Ref. [6]	
Propylene glycol, dipropylene glycol	Propylene oxide oxidation	Ref. [6]	
Propylene oxide	Chlorohydrin process	Ref. [6]	Technology chosen according to Kätelhön et al.
p-Xylene	Separation of xylenes by adsorption	Ref. [6]	
p-Xylene	Separation of xylenes by crystallization	Ref. [6]	
Pyrolysis gasoline	Steam cracking of naphtha	Ref. [6]	
Silicon carbide	Market for silicon carbide	Ref. [7]	
Sodium carbonate	Market for sodium bicarbonate	Ref. [7]	
Sodium chloride	Sodium chloride production, powder	Ref. [7]	
Steam	Natural gas boiler	Ref. [6]	
Styrene	Alkylation of benzene with ethylene (liquid-phase)	Ref. [6]	
Styrene	Alkylation of benzene with ethylene (gas-phase)	Ref. [6]	
Sulfur trioxide	Market for sulfur trioxide	Ref. [7]	
Sulfuric acid	Market for sulfuric acid	Ref. [7]	
Synthesis gas (2:1)	Natural gas steam reforming	Ref. [6]	
Thermal energy	Various hydrocarbons	Own calculation	Net calorific value
Terephthalic acid	Oxidation of p-xylene	Ref. [6]	
Toluene	Solvent extraction from reformate	Ref. [6]	
Toluene	Solvent extraction from pyrolysis gasoline	Ref. [6]	
Toluene	Separation of xylenes by adsorption	Ref. [6]	
Toluene	Separation of xylenes by crystallization	Ref. [6]	
Toluene diisocyanate	Phosgenation of toluene	Ref. [6]	
Vinyl chloride	Ethylene chlorination and ethylene dichloride pyrolysis	Ref. [6]	
Xylenes, mixed	Solvent extraction from reformate	Ref. [6]	

Product	Technologies	Source	Comment
Xylenes, mixed	Solvent extraction from pyrolysis gasoline	Ref. [6]	

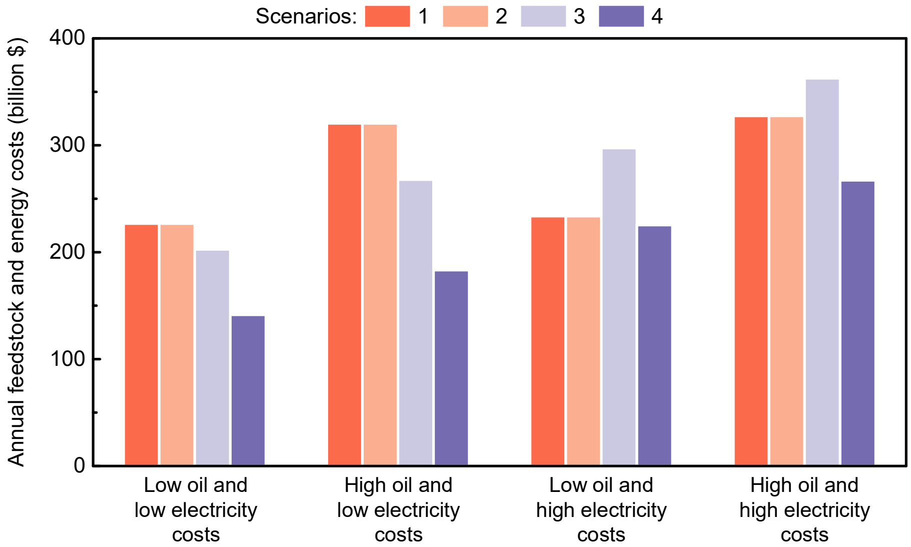

Supplementary Figure 7 Annual feedstock and energy costs of NH_3 manufacture and hydrocarbon-to-oxygenate conversions in four scenarios: (1) continuing to use fossil-based technologies, (2) using electricity only from renewable sources, without further changes to the manufacturing processes, (3) changing H_2 production over to renewable-energy-powered water electrolysis, and (4) the inclusion of renewable-energy-powered coupled electrolyzer technologies. The calculations are based on the price ranges of oil at $50 to 71 per barrel and electricity at 2 to 6 cents/kWh;[10] low oil/electricity costs are the lower bound of these ranges and high oil/electricity costs are the upper bound.

Supplementary References

1 Elvers, B. & Ullmann, F. *Ullmann's encyclopedia of industrial chemistry*. (Wiley-VCH,Weinheim, ed. 7, 2011).

2 Linstrom, P. J. M., William G. . *NIST Chemistry WebBook, NIST Standard Reference Database Number 69*. (NIST, 2018).

3 Ruscic, B. B., David H Active Thermochemical Tables (ATcT) values based on ver. 1.122 of the Thermochemical Network. *available at ATcT. anl. gov* (2019).

4 Kätelhön, A., Meys, R., Deutz, S., Suh, S. & Bardow, A. Climate change mitigation potential of carbon capture and utilization in the chemical industry. *Proc. Natl. Acad. Sci. U.S.A.* **116**, 11187 (2019).

5 Meys, R. *et al.* Achieving net-zero greenhouse gas emission plastics by a circular carbon economy. *Science* **374**, 71-76, doi:10.1126/science.abg9853 (2021).

6 Process Economics Program (PEP) Yearbook. (IHS Markit, Ed., 2018).

7 Wernet, G. *et al.* The ecoinvent database version 3 (part I): overview and methodology. *The International Journal of Life Cycle Assessment* **21**, 1218-1230, doi:10.1007/s11367-016-1087-8 (2016).

8 Matzen, M. J., Alhajji, M. H. & Demirel, Y. Technoeconomics and Sustainability of Renewable Methanol and Ammonia Productions Using Wind Power-based Hydrogen. (2015).

9 Agora, E. The Future Cost of Electricity-Based Synthetic Fuels. *Report* (2018).

10 IEA. Energy Technology Perspectives 2020. (https://www.iea.org/reports/energy-technology-perspectives-2020, 2020).
